# Identification of uncultured bacteria from abscesses of exotic pet animals using broad-range nested 16S rRNA polymerase chain reaction and Sanger sequencing

**DOI:** 10.14202/vetworld.2019.1546-1553

**Published:** 2019-10-09

**Authors:** T. Duangurai, J. Siengsanan-Lamont, C. Bumrungpun, G. Kaewmongkol, L. Areevijittrakul, T. Sirinarumitr, S. G. Fenwick, S. Kaewmongkol

**Affiliations:** 1 Department of Companion Animal Clinical Sciences Faculty of Veterinary Medicine, Kasetsart University, Bangkok, Thailand; 2 Private Consultant, 6 Gundaring Turn, Caning Vale, Western Australia 6155; 3 Exotic Pet Clinic, Faculty of Veterinary Medicine, Veterinary Teaching Hospital, Kasetsart University, Bangkok, Thailand; 4 Department of Pathology, Faculty of Veterinary Medicine, Kasetsart University, Bangkok, Thailand; 5 Department of Infectious Diseases and Global Health, Cummings School of Veterinary Medicine, Tufts University, Boston, MA, USA; 6 Department of Veterinary Technology, Faculty of Veterinary Technology, Kasetsart University, Bangkok, Thailand.

**Keywords:** anaerobic bacteria, abscesses, exotic pet animals, Sanger sequencing

## Abstract

**Background:**

The Sanger sequencing technique has been questioned and challenged by advanced high-throughput sequencing approaches. Sanger sequencing seems to be an obsolete technology. However, there are still research problems that could be answered using the Sanger sequencing technology. Fastidious obligate anaerobic bacteria are mostly associated with abscesses in animals. These bacteria are difficult to isolate from abscesses and are frequently excluded due to the bias of conventional bacterial culturing.

**Aim:**

This study demonstrated the usefulness of a broad-range polymerase chain reaction (PCR) with Sanger sequencing to identify the majority population of bacteria in abscesses from exotic pet animals.

**Materials and Methods:**

This study performed a pilot investigation of abscesses from 20 clinical cases (17 rabbits, 2 hedgehogs, and 1 sugar glider) using standard culture methods for both aerobes and anaerobes and broad-range nested PCR targeting the 16S rRNA gene followed by the Sanger sequencing technique.

**Results:**

The standard culture and PCR techniques detected bacteria in 9 and 17 of 20 samples, respectively. From the 17 sequencings of the 16S rRNA, 10 PCR products were found to be closely related with obligate anaerobes including *Bacteroides* spp., *Fusobacterium* spp., *Prevotella* spp. Phylogenetic analysis using the *rpoB* gene revealed that the species for the *Bacteroides* was *thetaiotaomicron* and for the *Fusobacterium* was *varium* and *nucleatum*. However, the amplification of the *rpoB* gene for the *Prevotella* spp. was unsuccessful. Correlations between the standard culture and PCR techniques were found in 9 (6 positive and 3 negative samples) of 20 samples. Eleven samples were discordant between the standard culture and PCR techniques which were composed of eight samples negative by culture but positive by PCR and three samples had different bacteria by the culture and PCR techniques.

**Conclusion:**

According to this study, broad-range PCR combined with Sanger sequencing might be useful for the detection of dominant anaerobic bacteria in abscesses that were overlooked based on conventional bacterial culture.

## Introduction

Broad-range polymerase chain reaction (PCR) detections have long been used for the studies of microorganism diversity within complex environments. In the past decades, these PCR and Sanger sequencing techniques have discovered large numbers of novel bacterial species and their ecosystems including soil, water, and plant as the main habitats of these novel species [1]. These broad-range PCRs targeting the 16S rRNA gene have also altered routine diagnosis in clinical microbiology as conventional bacteriological techniques have failed to identify some groups of organisms known as culture-negative bacteria [2]. Using Sanger and high-throughput sequencings of the 16S rRNA regions, polymicrobial populations of bacteria have been commonly detected in a part of inflamed specimens in humans [3-6]. Consequently, huge numbers of novel pathogens and high microbial diversity have also been revealed in human specimens using these broad-range PCRs followed by Sanger or high-throughput sequencing technologies that have opened a new era of the study of bacterial infections in human medicine [5,6]. Sterile inflammation or any inhibitory effects due to antibiotic treatments before sample collections have generally explained the culture-negative results in clinical specimens [2]. Eventually, the identification of bacterial diversity in culture-negative samples using high-throughput sequencing technologies changed previous perceptions of bacterial infections as causes of inflammatory diseases.

An abscess caused by bacterial infections is commonly diagnosed in exotic pet animals in Thailand, including rabbits, hedgehogs, and sugar gliders. Fastidious obligate anaerobic bacteria are mostly associated with abscesses in rabbits [7,8]. However, these bacteria are difficult to isolate from abscesses and are frequently excluded by the bias of conventional bacterial culture [9]. Although bacterial isolations directly from clinical specimens are routinely performed to identify anaerobic bacteria in abscesses, the culture-negative results may be due to errors in specimen preservation and transportation before the isolation process [10]. Microbial diversity in periodontal diseases in canines and ovines has been reported [11,12]. Genetic characterization of those predominant anaerobic bacteria has been accomplished using PCR techniques. The predominant anaerobic bacteria were also identified in bovines with periodontitis using the 16S Illumina platform next-generation DNA sequencing [13]. Without appropriate antimicrobial therapy, surgical drainage alone usually fails to treat abscesses filled with caseous pus and covered by a thick wall in those animals. Isolation of anaerobes is time consuming, expensive and requires highly experienced technicians.

PCR technique using universal primers for the bacterial 16S rRNA gene has been developed and PCR products have been sequenced using Sanger technology and blasted to determine the genus and species of these bacteria. This pilot study demonstrated the usefulness of a broad-range PCR with Sanger sequencing to identify the majority population of bacteria in abscesses from exotic pet animals. This report of anaerobic bacteria infection in exotic pet animals made veterinary practitioners reconsider the diagnostic techniques and treatment protocols for those abscesses and this report also demonstrates the advantage of the simple and cheap PCR technique combined with the Sanger sequencing to be an alternative approach.

## Materials and Methods

### Ethical approval

The leftover specimens were from the conventional bacterial culture in the routine work of Kasetsart University, Veterinary Teaching Hospital.

### Patients

We examined abscesses from 20 clinical cases at the exotic pet clinic, Kasetsart University Veterinary Teaching Hospital, Bangkok, Thailand (Table-1). The specimens in this study were collected using surgical removal.

**Table 1:** Classification of the PCR results compared with conventional culture results from 20 cases of abscesses.

Abscess site	Patient	Standard culture	DNA sequencing
Abdominal cavity	Rabbit	*Pseudomonas* sp.	*Pseudomonas aeruginosa*
Left tarsal joint	Rabbit	*Pseudomonas* spp.	*Pseudomonas aeruginosa*
Left lower eyelid	Rabbit	*Pseudomonas* spp.	*Pseudomonas aeruginosa*
Left ear pinna	Rabbit	*Pseudomonas* spp.	*Pseudomonas aeruginosa*
Tooth root	Rabbit	*Pasteurella multocida*	*Pasteurella multocida*
Tooth root	Rabbit	*Pseudomonas* spp.	*Pseudomonas aeruginosa*
Tooth root	Rabbit	No growth	PCR negative
Interdigital area	Rabbit	No growth	PCR negative
Left tarsal joint	Rabbit	No growth	PCR negative
Tooth root	Sugar glider	No growth	*Bacteroides fragilis*
Tooth root	Rabbit	No growth	*Bacteroides fragilis*
Abdominal cavity	Hedgehog	No growth	*Fusobacterium varium*
Tooth root	Rabbit	No growth	*Fusobacterium varium*
Tooth root	Rabbit	No growth	*Prevotella* spp.
Subcutaneous	Rabbit	No growth	*Fusobacterium varium*
Tooth root	Rabbit	No growth	*Fusobacterium nucleatum*
Subcutaneous	Hedgehog	β-*Streptococcus*	*Bacteroides fragilis*
Tooth root	Rabbit	*E. coli*	*Fusobacterium nucleatum*
Retrobulbar	Rabbit	*E. coli*	*Bacteroides massiliensis*
Tooth root	Rabbit	No growth	*E. coli*

*E. coli*=*Escherichia coli*, PCR=Polymerase chain reaction

### Conventional bacterial culture

Aerobic bacteria were cultured on MacConkey and 5% sheep blood agar plates (Merck, Darmstadt, Germany) incubated at 37°C for 24-48 h under aerobic conditions. Anaerobe bacterial isolation was performed using the GasPak procedure [14]. Conventional bacterial isolations were processed according to the standard protocol of the Bacteriology Laboratory, Kasetsart University Veterinary Teaching Hospital.

### DNA extraction and PCR detection

DNA was extracted from the contents inside the abscess using an E.Z.N.A.^®^ Tissue DNA Kit according to the manufacturer’s instructions. The nested PCR of 16S rRNA performed in this study was modified from the previous studies [15,16]. The *rpoB* loci were selected as the housekeeping gene targets for genetic analysis of those bacteria. The *rpoB* primers for anaerobic bacteria including *Bacteroides* spp., *Prevotella* spp., and *Fusobacterium* spp. were modified from the previous studies [17-19]. The *rpoB*-specific primers for *Pseudomonas* spp. were also used according to the previous study [20].

### DNA sequencing and phylogenetic analysis

An UltraClean^™^ 15 DNA Purification Kit was used for the purification of PCR products from the agarose gel. DNA sequencing was performed using an ABI Prism^™^ Terminator Cycle Sequencing Kit in an Applied Biosystem 3730 DNA Analyzer, according to the manufacturer’s instructions. Expected protein sequences of the *rpoB* gene were assembled using the BioEdit software Version 5.0.9 (http://www.mbio.nesu.edu/BioEdit).

Phylogenetic analyses of the nucleotide sequences of all loci were performed using the distance and maximum likelihood methods. Mega version 6 (Mega6: Molecular Evolutionary Genetics Analysis software, Arizona State University, Tempe, Arizona, USA) was used for phylogenetic analyses of the amino acid sequences of the *rpoB* protein of the *Fusobacterium* spp.

## Results

Bacterial DNA was detected using PCR technique in 17 specimens. From the 17 sequencings, 10 PCR products revealed that these bacteria had close relationships to obligate anaerobes, including *Bacteroides* spp. (two rabbits, a hedgehog, and a sugar glider), *Fusobacterium* spp. (four rabbits and a hedgehog), and *Prevotella* spp. (a rabbit). A housekeeping gene, the *rpoB* gene, of these anaerobes, was amplified. The BLAST results and phylogenetic analyses of the *rpoB* gene demonstrated the species status of *Bacteroides thetaiotaomicron*, *Fusobacterium varium*, and *Fusobacterium nucleatum*. Amplification of the *Prevotella* spp.’s *rpoB* gene was unsuccessful. Five PCR-positive samples were closely related to *Pseudomonas* spp. and phylogenetic analyses of the *rpoB* gene demonstrated *Pseudomonas aeruginosa*. The sequencing results of the 16S rRNA gene revealed that the other two specimens from rabbits were *Pasteurella* spp. and *Escherichia coli*. Correlations between the standard culture and PCR techniques were found in all five samples of *P. aeruginosa* and a sample of *Pasteurella* spp. In total, 11 (of 20) samples were cultured negative. Three of these culture-negative samples were also negative for PCR detection. *E. coli* and β-*Streptococcus* were isolated from two and one specimens, respectively, using standard culture, which were results discordant with PCR results. The results of these 20 cases were classified into four groups: (1) Agreement between the results of the conventional culture and PCR techniques, (2) negative results from both techniques, (3) negative culture with positive PCR for anaerobes, and (4) discordant results.

**Figure-1:**
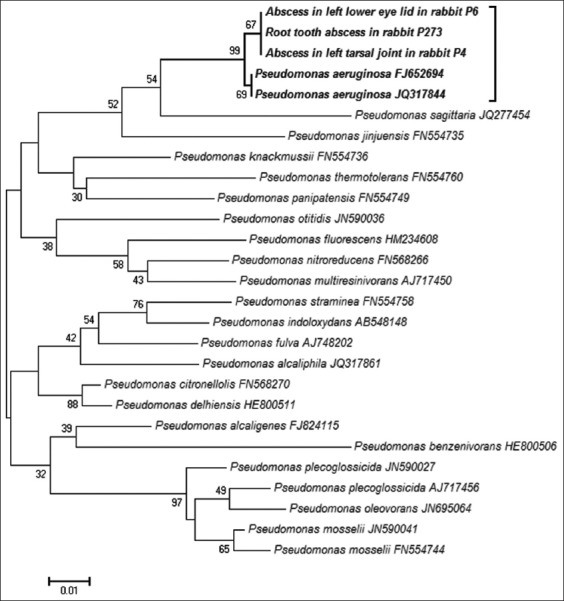
Neighbor-joining phylogenetic tree of the *rpoB* gene of *Pseudomonas* species detected in the abscesses (GenBank accession number; MG917661-MG917663) and validated genotypes of *Pseudomonas* species.

*Pseudomonas* spp. is known to be one of the most common bacteria causing abscesses in rabbits. [21]. Our investigation found that five of the *Pseudomonas* spp. isolates from rabbit specimens were positive using both PCR and conventional culture methods (Table-1). Genetic analysis of the *rpoB* gene confirmed the species status of *Pseudomonas aeruginosa*. A neighbor-joining phylogenetic tree of the *rpoB* gene revealed that the *Pseudomonas aeruginosa* detected in this study was closely related to other validated genotypes reported in GenBank (98.5-99.1% identity) (Figure-1). One rabbit tooth root abscess sample was positive to *Pasteurella multocida* based on both techniques.

**Figure-2:**
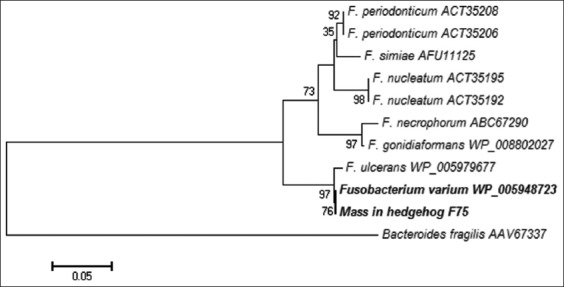
Maximum likelihood tree of the rpoB protein (RNA polymerase beta-subunit) sequences from *Fusobacterium* spp. (GenBank accession number; MG917664). Percentage bootstrap support from 1000 pseudoreplicates is indicated to the left of the supported node.

**Figure-3:**
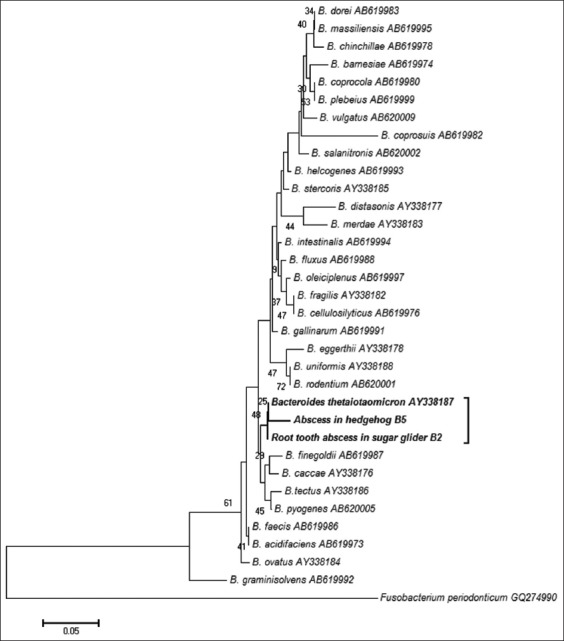
Neighbor-joining phylogenetic tree of the *rpoB* gene of *Bacteroides* spp. detected in the abscesses and validated genotypes of *Bacteroides* spp. (GenBank accession number; MG917665-MG917666). Percentage bootstrap support from 1000 pseudoreplicates is indicated to the left of the supported node.

Of the 20 cases, PCR test detected bacterial DNA in 17 specimens with three negative results. When compared to the conventional bacterial culture technique, only nine specimens were positive. Seven of 11 culture-negative samples were positive based on PCR technique. Three specimens that were negative to both techniques may not have been caused by bacterial infections. As the majority of specimens in this study were from tooth root abscesses, the anaerobes isolated most likely originated from periodontal bacteria (Table-1). Maximum likelihood and neighbor-joining phylogenetic trees of the *rpoB* gene confirmed that the bacterial species were *Fusobacterium* spp. and *Bacteroides* spp. (Figures-2 and 3). Aerobic bacteria isolated using the conventional bacteria culture included β-*Streptococcus* spp. and *E. coli*; however, the culture results were discordant with the nested PCR technique.

## Discussion

Bacterial ecology of intra-abdominal abscesses has been studied in humans using the 16S rRNA Illumina Sequencing technique [6]. In this report, the deep sequencing technique revealed that the organisms isolated using conventional culture were not the most dominant operational taxonomic units (OTUs) and concluded that the culturable bacteria were only minority populations. In addition, low microbial richness and diversity were demonstrated in Gram-stain/culture-negative samples. Reductions in the overall microbial richness and ecological diversity were also reported in interstitial cystitis, the chronic inflammation state of the urinary bladder in women compared with microbial profiles in the urine of healthy women using 16S rRNA PCR and pyrosequencing [22]. Sanger sequencing technology was confirmed to retrieve most abundant Illumina OTU in the study of photobiont diversity in lichens [23]. It has been proposed that the dominant organisms in each ecological niche might be the key species in engineering the environment and manipulating the biotic interactions rather than the minority species [23-25]. In our study, most bacterial species detected in culture-negative samples using Sanger sequencing were obligate anaerobes. Therefore, it was highly likely that these anaerobes were the dominant pathogenic bacteria in the culture-negative samples. These findings in our study emphasized the ability of 16S PCR with Sanger technology as a useful tool for pathogenic bacterial identification, particularly in culture-negative samples.

Bacterial infections commonly cause severe and chronic diseases in exotic small mammal pets [26]. Rabbits and rodents are more likely to form abscesses contained with caseous and purulent discharge than other exotic pets [27,28]. Infections can be caused by both Gram-positive and Gram-negative bacteria. Even though most infections are caused by aerobic bacteria, anaerobes could also cause disease. Bacteria could be isolated from several organs of ill animals [29].

Although the incidence of root teeth abscesses in pet rabbits is high, publications and research on the bacteriology of these infections are limited. Selecting suitable antibiotic for treatment is difficult as bacterial culture from clinical cases can be ambiguous. In 2002, Tyrrell *et al*. [7] reported that a bacteriology study of 12 isolates from rabbit mandibular and maxillary abscesses was *F. nucleatum*, *Prevotella heparinolytica*, *Prevotella* spp., *Peptostreptococcus micros*, *Streptococcus milleri* group, *Actinomyces israelii*, and *Arcanobacterium haemolyticum*. Similar to Tyrrell’s study [7], our investigation isolated *Fusobacterium* spp. in rabbit specimens.

Rabbits have long been used as laboratory models for human gingivitis and periodontal disease studies. Anaerobic bacteria play an important role in infections and abscess formation [21]. Periapical infections and abscesses in rabbits and rodents caused by *P. multocida* have been reported [27,30]. In addition, other bacteria reported causing abscesses include *Staphylococcus aureus*, *Pseudomonas aeruginosa, Bacteroides* spp., and *Proteus* spp., either alone or in mixed infection [27,31]. Abscesses in rabbits are often related to underlying dental diseases [32]. Most rabbit cases that formed periapical and facial abscesses resulted in progressive osteomyelitis [32]. The treatment of choice is a combination of surgical removal of exudates, necrotic debris and infected tissue, and the administration of systemic antibiotics. According to patient records at the Kasetsart Veterinary Teaching Hospital between 2010 and 2012, 95 rabbits with root teeth and facial abscesses were outpatients at the exotic pet clinic (unpublished data). From these clinical cases, there were 125 isolates of 11 bacterial species including *E. coli*, alpha-*Streptococci*, *Klebsiella* spp., *Staphylococcus* spp., *Pseudomonas* spp., *Pasteurella* spp., *Proteus* spp., *Enterobacter* spp., *Moraxella* spp., *Actinobacillus* spp., and *Bacillus cereus*. Similar to our study, most aerobes isolated from these rabbit specimens were *Pseudomonas* spp. (46.4%). In addition, 92%, 86.4%, 72.8%, and 61.6% of the isolates were sensitive to amikacin, gentamicin, ciprofloxacin, and enrofloxacin, respectively, according to *in vitro* antibiotic sensitivity tests.

**Figure-4:**
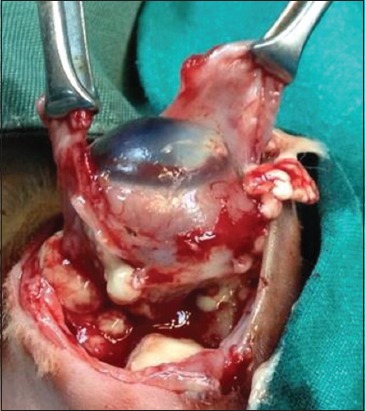
Surgical treatment of rabbit retrobulbar abscesses by enucleation and abscess drainage procedure.

One of the currently recommended antibiotics is fluoroquinolone which is commonly used in exotic pet medicine. Enrofloxacin is the preferred antibiotic to manage many bacterial infections in lagomorphs, for example, *P. multocida* and *Mycoplasma* spp. [33]. Retrobulbar abscesses in rabbits were often reported with a poor prognosis [34]. One rabbit included in this study presented with a retrobulbar abscess from a periapical origin (Figure-4).

Antimicrobial susceptibility testing (AST) is really needed for the decision-making for antibiotic treatments of bacterial infection cases. However, there are so many reports that have demonstrated some difficulties for growing of fastidious bacteria, for example, anaerobic bacteria and the alpha-*Proteobacteria* group. Our research team has experience of these alpha-*Proteobacteria* which are difficult to culture and most of these bacteria are well known as real pathogens in both animals and humans. We have worked and published these organisms including *Bartonella* spp., *Ehrlichia* spp., *Anaplasma* spp., and hemotropic *Mycoplasma* spp. that all of these bacteria have not been successfully grown by *in vitro* culture yet. Therefore, based on the current knowledge, ASTs for these bacteria are completely impossible. AST of these bacteria including anaerobes is strongly required proper and specific culture techniques. Consequently, identification of specific species of bacteria based on DNA detection has to be done before finding out complex or particular nutritional requirements for *in vitro* culture of these fastidious bacteria.

Treatment of choice for periapical and facial abscesses is a combination of a surgical therapy (to remove lesions, affected bone debridement, and associated teeth), drainage of abscesses, and appropriate analgesic and antimicrobial drug administration [34]. Enucleation is sometimes required to access the abscesses behind the eyeball. Success of abscess treatment depends on the following factors: Ability to define the extent of the lesion, especially those involving bones (maxilla and/or mandible), surgical accessibility to the abscess location, and continuity of care after operations. Ideally, excising the entire abscess capsule with marsupialization and packing the surgical site with polymethylmethacrylate beads mixed with the antibiotic of choice (based on the results of a sensitivity test) should be applied.

**Figure-5:**
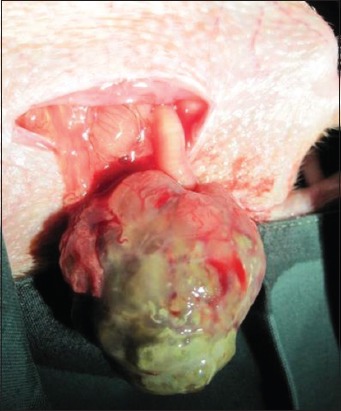
Surgical treatment of intra-abdominal abscess in hedgehog.

Even though aerobic and anaerobic culture and antibiotic sensitivity testing provide critical data that are essential for antibiotic selection, the conventional culture of anaerobic bacteria requires an experienced technician and specific methods (described above) and is difficult to process in routine work. In the current study, rabbit specimens that were positive to *Pseudomonas* spp. based on standard culture and DNA sequencing techniques were successfully treated using an antibiotic selected on the sensitivity test result. Culture-negative specimens may be due to possible factors, including previous antibiotic usage and/or wound management procedures [35]. In the current study, it was not possible to clarify the sources of bacterial infection of intra-abdominal abscesses the rabbits and hedgehog (Figure-5).

The discordant results in the current study revealed possible errors in both the anaerobe culture (used in a routine diagnosis) and the nested PCR technique (used to identify dominant bacteria in abscess specimens). Infection leading to abscess formation in exotic pets caused by anaerobes could be underestimated if only conventional culture testing is ­conducted. The new broad-range PCR technique demonstrated a benefit as an alternative tool which will help to confirm the results of conventional culture, particularly anaerobic bacterial culture. In summary, specimen transportation and anaerobic bacterial culture protocols need to be improved. Implantation of specific antibiotic beads for anaerobic bacteria into the surgical area after abscess removal could increase the treatment success rate [14].

## Conclusion

This study reports the simple broad-range PCR combined with Sanger sequencing that was able to detect dominant anaerobic bacteria in abscesses of exotic pets in Thailand. According to our experience as veterinary practitioners in this region, anaerobic bacteria were frequently overlooked by conventional bacterial culture. Treatment failure of abscesses in these animals could be caused by the negative results of anaerobic bacterial culture method. Therefore, anaerobic bacterial infections were excluded from the list of differential diagnosis and specific antibiotics for anaerobes were not used in these cases. This study performed the genetic characterization of anaerobes in clinical specimens collected from the abscesses of exotic pets that this genetic information has been very limited in this geographical area.

## Authors’ Contributions

TD and LA collected the specimens and reported the cases. CB performed bacterial culture both aerobes and anaerobes. GK and SK designed the experiment and made DNA extractions, PCRs, and multiple DNA sequences analyses. JS, TS, and SGF were involved in scientific discussion and provided suggestions for the overall work. All authors read and approved the final manuscript.
